# Crystal structure of dimethyl 9*H*-carbazole-2,7-di­carb­oxy­late

**DOI:** 10.1107/S2056989015017557

**Published:** 2015-09-26

**Authors:** Ryan L. Lehane, James A. Golen, Arnold L. Rheingold, David R. Manke

**Affiliations:** a Department of Chemistry and Biochemistry, University of Massachusetts Dartmouth, 285 Old Westport Road, North Dartmouth, MA 02747, USA; b Department of Chemistry, University of California, San Diego, 9500 Gilman Drive, La Jolla, CA 92093, USA

**Keywords:** crystal structure, carbazoles, hydrogen bonding, π–π inter­actions

## Abstract

In the title compound, C_16_H_13_NO_4_, the carbazole ring system is almost planar with non-H atoms possessing a mean deviation from planarity of 0.037 Å. The two ester groups are orientated *trans* to one another and tilted slightly from the mean plane of the carbazole ring system, making dihedral angles of 8.12 (6) and 8.21 (5)°. In the crystal, mol­ecules are linked by pairs of N—H⋯O hydrogen bonds forming inversion dimers. The dimers are linked by parallel slipped π–π inter­actions, forming slabs propagating along the *b-*axis direction [inter-centroid distance = 3.6042 (8) Å, inter-planar distance = 3.3437 (5) Å, slippage = 1.345 Å].

## Related literature   

For the synthesis of the title compound, see: Olkhovik *et al.* (2008 ▸). For the crystal structures of some carbazoles, see: Clarke & Spink (1969 ▸); Gajda *et al.* (2014 ▸). For the structure of 9*H*-carbazole-3,6-di­carb­oxy­lic acid, see: Weseliński *et al.* (2014 ▸). For coordination polymers featuring the di­carboxyl­ate of the parent compound, see: Yi *et al.* (2013 ▸, 2014 ▸, 2015 ▸). 
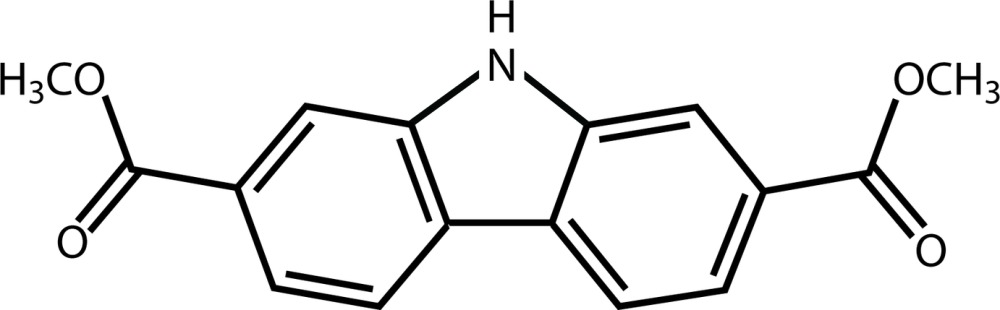


## Experimental   

### Crystal data  

C_16_H_13_NO_4_
*M* *_r_* = 283.27
Monoclinic, 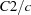

*a* = 29.684 (2) Å
*b* = 5.8264 (4) Å
*c* = 15.4210 (11) Å
β = 96.252 (3)°
*V* = 2651.2 (3) Å^3^
*Z* = 8
Mo *K*α radiation
μ = 0.10 mm^−1^
*T* = 100 K
0.28 × 0.18 × 0.10 mm

### Data collection  

Bruker CMOS detector diffractometer
Absorption correction: multi-scan (*SADABS*; Bruker, 2005 ▸) *T*_min_ = 0.972, *T*_max_ = 0.990
16278 measured reflections
2716 independent reflections
2141 reflections with *I* > 2σ(*I*)
*R*_int_ = 0.042

### Refinement  

*R*[*F* ^2^ > 2σ(*F* ^2^)] = 0.037
*wR*(*F* ^2^) = 0.098
*S* = 1.03
2716 reflections
195 parameters
1 restraint
H atoms treated by a mixture of independent and constrained refinement
Δρ_max_ = 0.22 e Å^−3^
Δρ_min_ = −0.22 e Å^−3^

### 

Data collection: *APEX2* (Bruker, 2005 ▸); cell refinement: *SAINT* (Bruker, 2005 ▸); data reduction: *SAINT*; program(s) used to solve structure: *SHELXS97* (Sheldrick, 2008 ▸); program(s) used to refine structure: *SHELXL97* (Sheldrick, 2008 ▸); molecular graphics: *SHELXTL* (Sheldrick, 2008 ▸); software used to prepare material for publication: *SHELXL97*, *PLATON* (Spek (2009 ▸) and *publCIF* (Westrip, 2010 ▸).

## Supplementary Material

Crystal structure: contains datablock(s) I, New_Global_Publ_Block. DOI: 10.1107/S2056989015017557/su5208sup1.cif

Structure factors: contains datablock(s) I. DOI: 10.1107/S2056989015017557/su5208Isup2.hkl

Supporting information file. DOI: 10.1107/S2056989015017557/su5208Isup3.cml

**Fig. 1.**
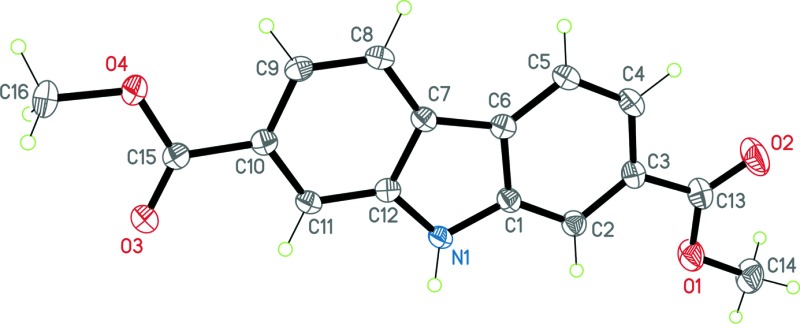
Molecular structure of the title compound, with atom labelling. Displacement ellipsoids are drawn at the 50% probability level.

**Fig. 2.**
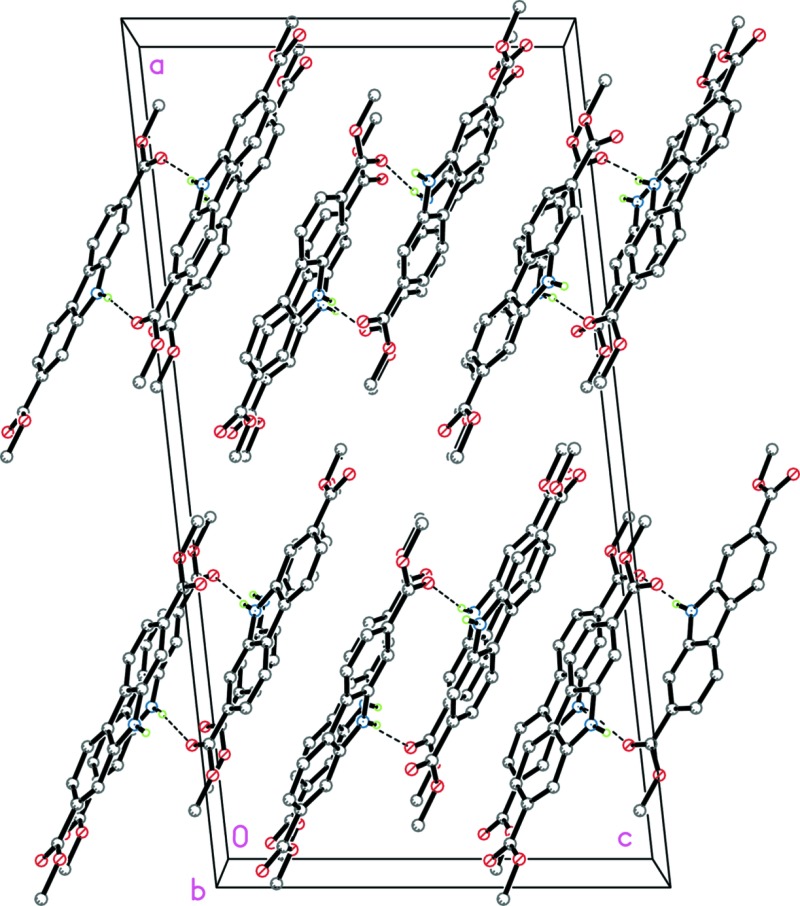
A view along the b axis of the crystal packing of the title compound, with hydrogen bonds shown as dashed lines (ee Table 1).

. DOI: 10.1107/S2056989015017557/su5208fig1.tif
Mol­ecular structure of the title compound, with atom labelling. Displacement ellipsoids are drawn at the 50% probability level.

b . DOI: 10.1107/S2056989015017557/su5208fig2.tif
A view along the *b* axis of the crystal packing of the title compound, with hydrogen bonds shown as dashed lines (ee Table 1).

CCDC reference: 1426074

Additional supporting information:  crystallographic information; 3D view; checkCIF report

## Figures and Tables

**Table 1:** Hydrogen-bond geometry (, )

*D*H*A*	*D*H	H*A*	*D* *A*	*D*H*A*
N1H1*N*O3^i^	0.87(1)	2.04(1)	2.8834(16)	164(1)

Symmetry code: (i) 
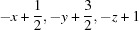
.

## supplementary crystallographic information

### S1. Comment

Bi­phenyl-4,4'-di­carboxyl­ate and its derivatives are widely used in metal-organic frameworks (MOFs) as linkers. One derivative that is being explored in coordination polymers is 9*H*-carbazole-2,7-di­carboxyl­ate (Yi *et al.*, 2013, 2014, 2015). Herein, we report on the crystal structure of the previously synthesized 9*H*-carbazole-2,7-di­carb­oxy­lic acid di­methyl ester (Olkhovik *et al.*, 2008).

The molecular structure of the title compound is shown in Fig. 1. The bond distances and angles are similar to those observed in some carbazole derivatives (Clarke & Spink, 1969; Gajda *et al.*, 2014,) and a structurally characterized carbazole di­carb­oxy­lic acid (Weseliński *et al.*, 2014). The carbazole unit is nearly planar with a mean deviation from planarity of 0.037 Å. The carboxyl­ate groups are *trans* to one another and skewed slightly from the mean plane of the carbazole unit, with carbazole-ester dihedral angles of 8.12 (6) and 8.21 (5)°, involving ester groups O1/O2/C3/C13/C14 and O3/O4/C10/C15/C16, respectively.

In the crystal, molecules are linked by a pair of N—H···O hydrogen bonds forming inversion dimers (Fig. 2 and Table 1). The dimers are linked by parallel slipped π-π inter­actions forming slabs propagating along the *b* axis direction [Cg3···Cg3^i^ = 3.6042 (8) Å, inter­planar distance = 3.3437 (5) Å, slippage 1.345 Å; Cg3 is the centroid of ring C7—C12; symmetry code: (i) -x+1/2, -y+1/2, -z+1].

### S2. Synthesis and crystallization

The compound was prepared by a literature procedure (Olkhovik *et al.* 2008). Crystals suitable for X-ray diffraction analysis were grown by slow evaporation of a solution in ethanol.

### S3. Refinement

Crystal data, data collection and structure refinement details are summarized in Table 2. Hydrogen atom H1N was located in a difference Fourier map and refined with a distance restraint: N—H = 0.87 (2) Å with U_iso_(H) = 1.2*U*_eq_(N). The C-bound H atoms were placed in calculated positions and refined as riding: C—H = 0.95-0.98 Å with U_iso_(H) =1.5*U*_eq_(C) for methyl H atoms and 1.2U_eq_(C) for other H atoms.

### Crystal data

**Table:**

C_16_H_13_NO_4_	*F*(000) = 1184
*M**_r_* = 283.27	*D*_x_ = 1.419 Mg m^−^^3^
Monoclinic, *C*2/*c*	Mo *K*α radiation, λ = 0.71073 Å
Hall symbol: -C 2yc	Cell parameters from 5867 reflections
*a* = 29.684 (2) Å	θ = 3.1–26.3°
*b* = 5.8264 (4) Å	µ = 0.10 mm^−^^1^
*c* = 15.4210 (11) Å	*T* = 100 K
β = 96.252 (3)°	Block, yellow
*V* = 2651.2 (3) Å^3^	0.28 × 0.18 × 0.10 mm
*Z* = 8	

### Data collection

**Table:**

Bruker CMOS detector diffractometer	2716 independent reflections
Radiation source: fine-focus sealed tube	2141 reflections with *I* > 2σ(*I*)
Doubly curved mirrors monochromator	*R*_int_ = 0.042
φ and ω scans	θ_max_ = 26.4°, θ_min_ = 2.7°
Absorption correction: multi-scan (*SADABS*; Bruker, 2005)	*h* = −36→36
*T*_min_ = 0.972, *T*_max_ = 0.990	*k* = −7→7
16278 measured reflections	*l* = −19→19

### Refinement

**Table:**

Refinement on *F*^2^	Primary atom site location: structure-invariant direct methods
Least-squares matrix: full	Secondary atom site location: difference Fourier map
*R*[*F*^2^ > 2σ(*F*^2^)] = 0.037	Hydrogen site location: inferred from neighbouring sites
*wR*(*F*^2^) = 0.098	H atoms treated by a mixture of independent and constrained refinement
*S* = 1.03	*w* = 1/[σ^2^(*F*_o_^2^) + (0.0509*P*)^2^ + 1.5495*P*] where *P* = (*F*_o_^2^ + 2*F*_c_^2^)/3
2716 reflections	(Δ/σ)_max_ = 0.001
195 parameters	Δρ_max_ = 0.22 e Å^−^^3^
1 restraint	Δρ_min_ = −0.22 e Å^−^^3^

### Special details

**Table:**

Geometry. All e.s.d.'s (except the e.s.d. in the dihedral angle between two l.s. planes) are estimated using the full covariance matrix. The cell e.s.d.'s are taken into account individually in the estimation of e.s.d.'s in distances, angles and torsion angles; correlations between e.s.d.'s in cell parameters are only used when they are defined by crystal symmetry. An approximate (isotropic) treatment of cell e.s.d.'s is used for estimating e.s.d.'s involving l.s. planes.
Refinement. Refinement of *F*^2^ against ALL reflections. The weighted *R*-factor *wR* and goodness of fit *S* are based on *F*^2^, conventional *R*-factors *R* are based on *F*, with *F* set to zero for negative *F*^2^. The threshold expression of *F*^2^ > σ(*F*^2^) is used only for calculating *R*-factors(gt) *etc*. and is not relevant to the choice of reflections for refinement. *R*-factors based on *F*^2^ are statistically about twice as large as those based on *F*, and *R*- factors based on ALL data will be even larger.

### Fractional atomic coordinates and isotropic or equivalent isotropic displacement parameters (Å^2^)

**Table:**

	*x*	*y*	*z*	*U*_iso_*/*U*_eq_	
O1	0.46191 (4)	0.66773 (19)	0.82835 (7)	0.0330 (3)	
O2	0.47499 (4)	0.3231 (2)	0.88997 (8)	0.0435 (3)	
O3	0.15036 (3)	0.56249 (18)	0.46010 (7)	0.0281 (3)	
O4	0.12301 (3)	0.23787 (18)	0.51083 (7)	0.0287 (3)	
N1	0.31090 (4)	0.5801 (2)	0.63776 (8)	0.0206 (3)	
H1N	0.3176 (5)	0.703 (2)	0.6095 (10)	0.025*	
C1	0.34155 (4)	0.4608 (2)	0.69505 (8)	0.0193 (3)	
C2	0.38361 (5)	0.5315 (2)	0.73472 (9)	0.0214 (3)	
H2A	0.3958	0.6769	0.7221	0.026*	
C3	0.40714 (5)	0.3814 (2)	0.79351 (9)	0.0224 (3)	
C4	0.38949 (5)	0.1642 (3)	0.81051 (10)	0.0250 (3)	
H4A	0.4066	0.0629	0.8496	0.030*	
C5	0.34764 (5)	0.0959 (2)	0.77109 (9)	0.0217 (3)	
H5A	0.3358	−0.0508	0.7831	0.026*	
C6	0.32293 (5)	0.2456 (2)	0.71332 (9)	0.0185 (3)	
C7	0.27864 (5)	0.2357 (2)	0.66416 (8)	0.0179 (3)	
C8	0.24369 (5)	0.0734 (2)	0.65636 (9)	0.0196 (3)	
H8A	0.2472	−0.0684	0.6868	0.024*	
C9	0.20400 (5)	0.1208 (2)	0.60415 (9)	0.0195 (3)	
H9A	0.1802	0.0107	0.5982	0.023*	
C10	0.19859 (4)	0.3317 (2)	0.55960 (9)	0.0190 (3)	
C11	0.23263 (4)	0.4955 (2)	0.56654 (9)	0.0189 (3)	
H11A	0.2287	0.6376	0.5365	0.023*	
C12	0.27267 (4)	0.4461 (2)	0.61861 (9)	0.0183 (3)	
C13	0.45135 (5)	0.4484 (3)	0.84215 (10)	0.0271 (3)	
C14	0.50435 (5)	0.7491 (3)	0.87314 (12)	0.0371 (4)	
H14A	0.5076	0.9135	0.8620	0.056*	
H14B	0.5296	0.6655	0.8518	0.056*	
H14C	0.5046	0.7233	0.9360	0.056*	
C15	0.15579 (5)	0.3916 (2)	0.50484 (9)	0.0205 (3)	
C16	0.08012 (5)	0.2876 (3)	0.46017 (11)	0.0348 (4)	
H16A	0.0590	0.1609	0.4661	0.052*	
H16B	0.0849	0.3058	0.3986	0.052*	
H16C	0.0675	0.4298	0.4815	0.052*	

### Atomic displacement parameters (Å^2^)

**Table:**

	*U*^11^	*U*^22^	*U*^33^	*U*^12^	*U*^13^	*U*^23^
O1	0.0263 (6)	0.0291 (6)	0.0403 (7)	−0.0047 (5)	−0.0108 (5)	0.0021 (5)
O2	0.0376 (7)	0.0349 (7)	0.0522 (8)	0.0020 (5)	−0.0209 (6)	0.0067 (6)
O3	0.0248 (6)	0.0295 (6)	0.0291 (6)	−0.0003 (4)	−0.0013 (4)	0.0094 (5)
O4	0.0209 (5)	0.0290 (6)	0.0346 (6)	−0.0051 (4)	−0.0045 (5)	0.0051 (5)
N1	0.0205 (6)	0.0176 (6)	0.0229 (6)	−0.0017 (5)	−0.0012 (5)	0.0058 (5)
C1	0.0210 (7)	0.0189 (7)	0.0182 (7)	0.0032 (5)	0.0024 (5)	0.0010 (5)
C2	0.0213 (7)	0.0200 (7)	0.0227 (7)	0.0007 (6)	0.0014 (6)	0.0001 (6)
C3	0.0219 (7)	0.0236 (7)	0.0212 (7)	0.0026 (6)	0.0003 (6)	−0.0012 (6)
C4	0.0276 (8)	0.0240 (7)	0.0225 (7)	0.0054 (6)	−0.0008 (6)	0.0038 (6)
C5	0.0267 (8)	0.0182 (7)	0.0201 (7)	0.0012 (6)	0.0022 (6)	0.0022 (6)
C6	0.0218 (7)	0.0182 (7)	0.0157 (7)	0.0007 (5)	0.0035 (5)	−0.0022 (5)
C7	0.0219 (7)	0.0187 (7)	0.0135 (6)	0.0025 (5)	0.0040 (5)	−0.0010 (5)
C8	0.0253 (7)	0.0163 (6)	0.0180 (7)	0.0006 (5)	0.0053 (6)	0.0011 (5)
C9	0.0219 (7)	0.0175 (7)	0.0198 (7)	−0.0027 (5)	0.0046 (6)	−0.0025 (5)
C10	0.0206 (7)	0.0200 (7)	0.0167 (7)	0.0002 (6)	0.0036 (5)	−0.0017 (6)
C11	0.0222 (7)	0.0165 (6)	0.0182 (7)	0.0013 (5)	0.0034 (5)	0.0020 (5)
C12	0.0204 (7)	0.0173 (6)	0.0173 (6)	−0.0009 (5)	0.0032 (5)	−0.0010 (5)
C13	0.0265 (8)	0.0267 (8)	0.0270 (8)	0.0041 (6)	−0.0025 (6)	−0.0021 (6)
C14	0.0266 (9)	0.0372 (9)	0.0446 (10)	−0.0064 (7)	−0.0093 (7)	−0.0028 (8)
C15	0.0212 (7)	0.0213 (7)	0.0192 (7)	−0.0016 (6)	0.0031 (6)	−0.0015 (6)
C16	0.0218 (8)	0.0428 (10)	0.0374 (9)	−0.0036 (7)	−0.0068 (7)	0.0025 (8)

### Geometric parameters (Å, º)

**Table:**

O1—C13	1.3382 (19)	C5—H5A	0.9500
O1—C14	1.4490 (18)	C6—C7	1.445 (2)
O2—C13	1.2070 (18)	C7—C8	1.3992 (19)
O3—C15	1.2119 (17)	C7—C12	1.4145 (19)
O4—C15	1.3333 (17)	C8—C9	1.381 (2)
O4—C16	1.4487 (18)	C8—H8A	0.9500
N1—C12	1.3824 (17)	C9—C10	1.409 (2)
N1—C1	1.3842 (17)	C9—H9A	0.9500
N1—H1N	0.870 (13)	C10—C11	1.3852 (19)
C1—C2	1.3915 (19)	C10—C15	1.4884 (19)
C1—C6	1.4112 (19)	C11—C12	1.3902 (19)
C2—C3	1.391 (2)	C11—H11A	0.9500
C2—H2A	0.9500	C14—H14A	0.9800
C3—C4	1.406 (2)	C14—H14B	0.9800
C3—C13	1.491 (2)	C14—H14C	0.9800
C4—C5	1.381 (2)	C16—H16A	0.9800
C4—H4A	0.9500	C16—H16B	0.9800
C5—C6	1.3963 (19)	C16—H16C	0.9800
			
C13—O1—C14	116.30 (12)	C8—C9—H9A	119.9
C15—O4—C16	115.68 (12)	C10—C9—H9A	119.9
C12—N1—C1	108.67 (11)	C11—C10—C9	121.32 (13)
C12—N1—H1N	125.6 (10)	C11—C10—C15	116.91 (12)
C1—N1—H1N	124.2 (10)	C9—C10—C15	121.75 (12)
N1—C1—C2	128.90 (13)	C10—C11—C12	118.22 (12)
N1—C1—C6	109.20 (12)	C10—C11—H11A	120.9
C2—C1—C6	121.84 (12)	C12—C11—H11A	120.9
C1—C2—C3	117.59 (13)	N1—C12—C11	129.57 (12)
C1—C2—H2A	121.2	N1—C12—C7	109.14 (12)
C3—C2—H2A	121.2	C11—C12—C7	121.27 (12)
C2—C3—C4	121.10 (13)	O2—C13—O1	122.98 (14)
C2—C3—C13	121.01 (13)	O2—C13—C3	124.78 (14)
C4—C3—C13	117.87 (13)	O1—C13—C3	112.24 (12)
C5—C4—C3	120.86 (13)	O1—C14—H14A	109.5
C5—C4—H4A	119.6	O1—C14—H14B	109.5
C3—C4—H4A	119.6	H14A—C14—H14B	109.5
C4—C5—C6	119.08 (13)	O1—C14—H14C	109.5
C4—C5—H5A	120.5	H14A—C14—H14C	109.5
C6—C5—H5A	120.5	H14B—C14—H14C	109.5
C5—C6—C1	119.49 (13)	O3—C15—O4	122.58 (13)
C5—C6—C7	133.96 (13)	O3—C15—C10	124.62 (13)
C1—C6—C7	106.53 (12)	O4—C15—C10	112.79 (12)
C8—C7—C12	119.44 (13)	O4—C16—H16A	109.5
C8—C7—C6	134.08 (13)	O4—C16—H16B	109.5
C12—C7—C6	106.46 (12)	H16A—C16—H16B	109.5
C9—C8—C7	119.52 (13)	O4—C16—H16C	109.5
C9—C8—H8A	120.2	H16A—C16—H16C	109.5
C7—C8—H8A	120.2	H16B—C16—H16C	109.5
C8—C9—C10	120.24 (13)		
			
C12—N1—C1—C2	−177.05 (13)	C8—C9—C10—C15	−178.18 (12)
C12—N1—C1—C6	0.08 (15)	C9—C10—C11—C12	0.28 (19)
N1—C1—C2—C3	177.00 (13)	C15—C10—C11—C12	178.78 (12)
C6—C1—C2—C3	0.2 (2)	C1—N1—C12—C11	177.91 (13)
C1—C2—C3—C4	1.5 (2)	C1—N1—C12—C7	−0.13 (15)
C1—C2—C3—C13	−176.94 (13)	C10—C11—C12—N1	−178.41 (13)
C2—C3—C4—C5	−1.8 (2)	C10—C11—C12—C7	−0.58 (19)
C13—C3—C4—C5	176.68 (13)	C8—C7—C12—N1	178.58 (12)
C3—C4—C5—C6	0.4 (2)	C6—C7—C12—N1	0.13 (15)
C4—C5—C6—C1	1.3 (2)	C8—C7—C12—C11	0.35 (19)
C4—C5—C6—C7	−177.33 (14)	C6—C7—C12—C11	−178.10 (12)
N1—C1—C6—C5	−178.98 (12)	C14—O1—C13—O2	1.1 (2)
C2—C1—C6—C5	−1.6 (2)	C14—O1—C13—C3	179.87 (13)
N1—C1—C6—C7	−0.01 (14)	C2—C3—C13—O2	−175.23 (15)
C2—C1—C6—C7	177.37 (12)	C4—C3—C13—O2	6.3 (2)
C5—C6—C7—C8	0.6 (3)	C2—C3—C13—O1	6.0 (2)
C1—C6—C7—C8	−178.20 (14)	C4—C3—C13—O1	−172.48 (13)
C5—C6—C7—C12	178.69 (14)	C16—O4—C15—O3	0.0 (2)
C1—C6—C7—C12	−0.07 (14)	C16—O4—C15—C10	179.05 (12)
C12—C7—C8—C9	0.20 (19)	C11—C10—C15—O3	7.1 (2)
C6—C7—C8—C9	178.13 (14)	C9—C10—C15—O3	−174.35 (13)
C7—C8—C9—C10	−0.49 (19)	C11—C10—C15—O4	−171.85 (12)
C8—C9—C10—C11	0.3 (2)	C9—C10—C15—O4	6.65 (18)

### Hydrogen-bond geometry (Å, º)

**Table:**

*D*—H···*A*	*D*—H	H···*A*	*D*···*A*	*D*—H···*A*
N1—H1*N*···O3^i^	0.87 (1)	2.04 (1)	2.8834 (16)	164 (1)

Symmetry code: (i) −*x*+1/2, −*y*+3/2, −*z*+1.
