# Supplementary figures and images for: Crystal structure of dimethyl 9H-carbazole-2,7-di­carb­oxy­late
Source: Acta Crystallogr E Crystallogr Commun. 2015 Sep 26;71(Pt 10):o784–5. doi: 10.1107/S2056989015017557 (PMC4647360; doi:10.1107/S2056989015017557)

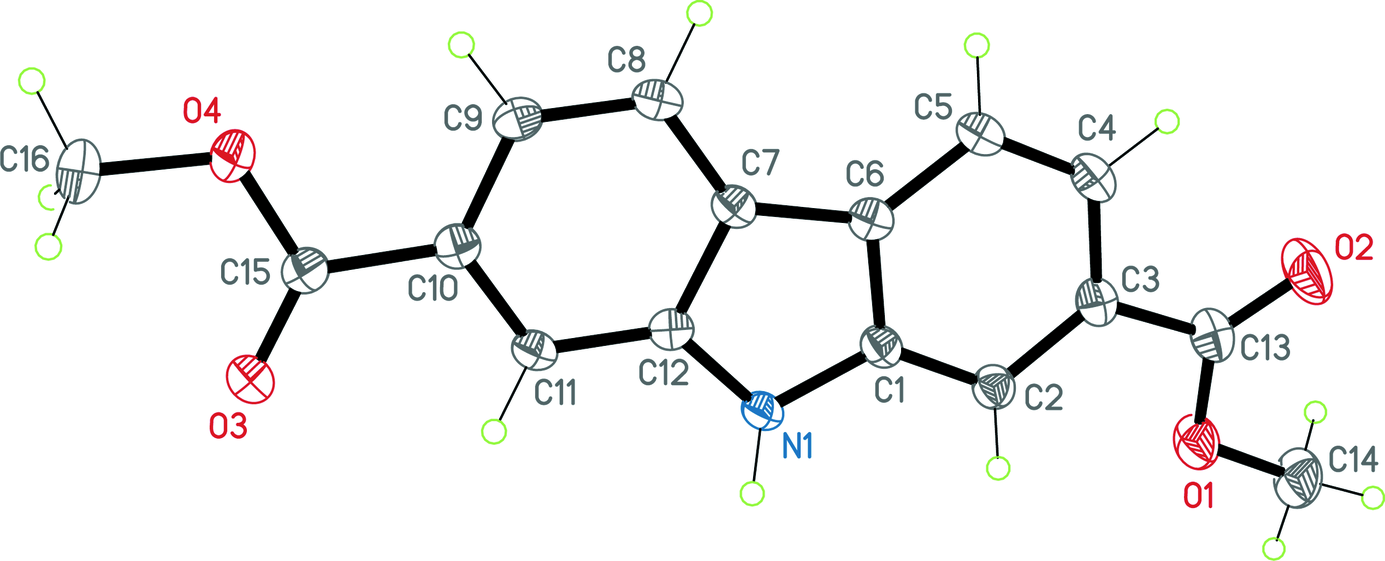

Supplement: Supplementary file 4 [file e-71-0o784-fig1.tif]

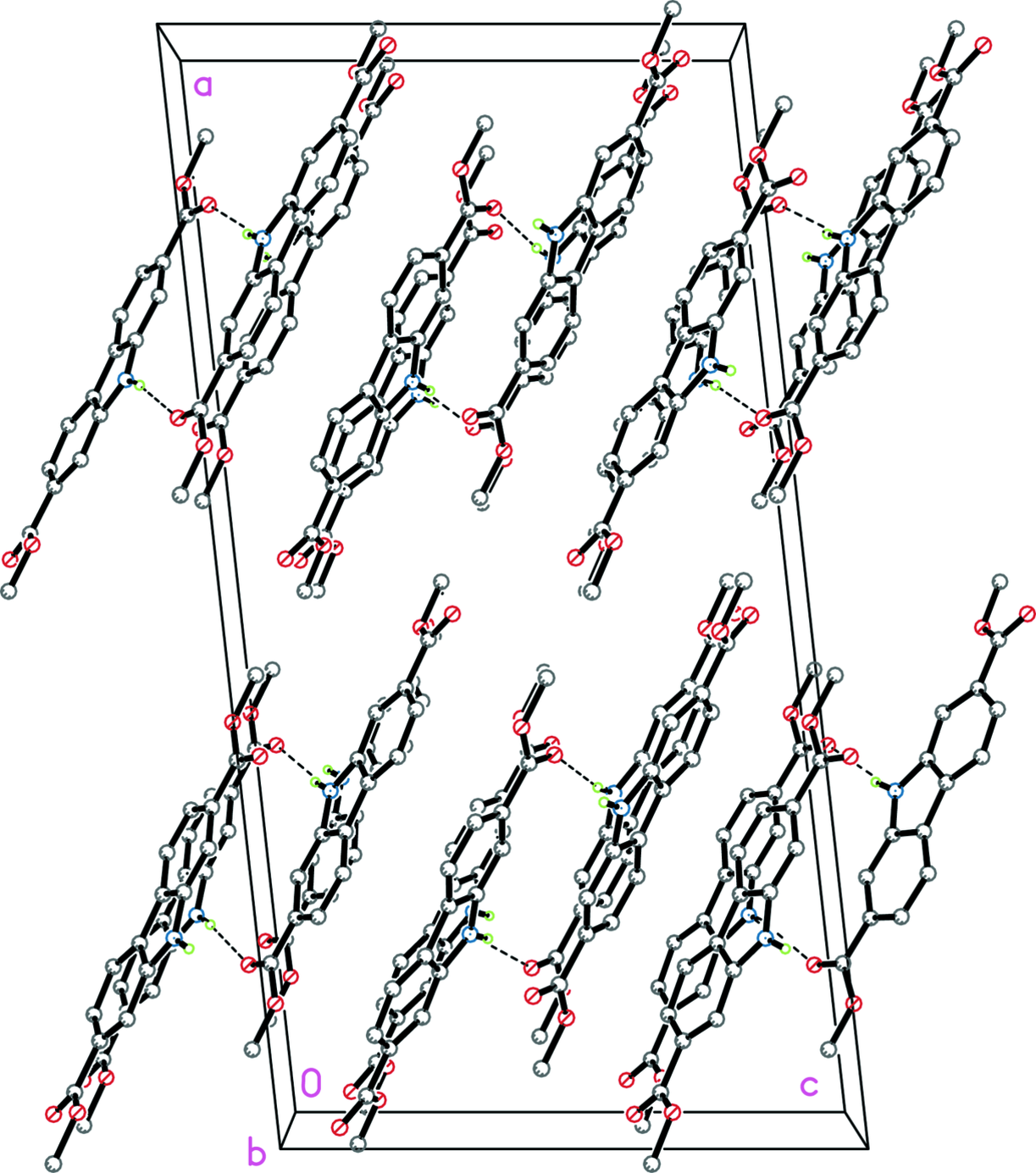

Supplement: Supplementary file 5 [file e-71-0o784-fig2.tif]
